# X-Linked Hypophosphatemia: Does Targeted Therapy Modify Dental Impairment?

**DOI:** 10.3390/jcm12247546

**Published:** 2023-12-07

**Authors:** Anusha Abdullah, Sabina Noreen Wuersching, Maximilian Kollmuss, Philipp Poxleitner, Ina Dewenter, Leonard Simon Brandenburg, David Steybe, Florian Nepomuk Fegg, Wenko Smolka, Sven Otto, Katharina Theresa Obermeier

**Affiliations:** 1 Department of Oral and Maxillofacial Surgery and Facial Plastic Surgery, Ludwig Maximilians University, 80337 Munich, Germany; 2 Department of Conservative Dentistry and Periodontology, University Hospital, LMU Munich, Goethestrasse 70, 80336 Munich, Germany; 3 Medical Center—University of Freiburg, Center for Dental Medicine, Department of Oral and Maxillofacial Surgery, Faculty of Medicine, University of Freiburg, Hugstetter Str. 55, 79106 Freiburg, Germany

**Keywords:** Burosumab, X-linked hypophosphatemia, dental abscesses, vitamin-D-resistant rickets

## Abstract

X-linked hypophosphatemia is a rare, hereditary disorder that significant influences teeth and alveolar bone. The first clinical sign leading to the diagnosis of X-linked hypophosphatemia is often dental impairment with dental abscesses and dentin mineralization defects. Genetic analysis helped find the responsible gene and therefore opened up new ways of therapeutically managing X-linked hypophosphatemia. The human monoclonal antibody Burosumab represents a milestone in the targeted therapy of this hereditary disease by directly addressing its pathophysiology. Targeted therapy has been shown to improve skeletal impairment, pain, and phosphate metabolism. However, the influence of this new therapy on dental impairment has only been addressed in a few recent studies with varying results. Therefore, in this review, we aim to summarize the dental phenotype and analyze the different treatment modalities with a focus on dental impairment.

## 1. Introduction

X-linked hypophosphatemia (XLH) is a rare, hereditary disease firstly described by Albright, Butler, and Bloomberg in 1937 and is characterized by hyperphosphaturia and hypophosphatemia resistant to treatment with normal doses of vitamin D [1]. Several different terms such as “Vitamin-D resistant rickets”, “familial hypophosphatemia”, and “phosphate diabetes” describe the same entity [2,3].

Vitamin D deficiency results in classical rickets, whereas other forms that cannot be treated with vitamin D are known as vitamin-D-resistant rickets [2]. Most importantly, XLH is the most common form of vitamin-D-resistant rickets, and its incidence is estimated to be about 1 in 20,000 to 25,000 births [2,4,5]. Although not many studies have investigated this, the prevalence of XLH is estimated to be between 1 in 20,000 and 1 in 60,000 individuals [6,7].

XLH is characterized by hypophosphatemia, elevated alkaline phosphatase levels, and normal serum calcium levels [2]. It is an X-linked dominant gene defect; therefore, affected women are heterozygous and affected men are hemizygous [2]. In women, less severe manifestations can be observed, which may be due to a gene dosage effect and protection by the additional X chromosome or hormonal impact [8,9]. Because phosphate is essential for the structure of bone and teeth, infants in particular need more phosphate than adults for growth [10].

As dental impairment is often the first clinical sign of XLH, early diagnosis of this disease is required for causative therapy and better prognosis [2]. Clinical manifestations start appearing from the age of 8 to 10 months, which might be due to the low glomerular filtration rate in infancy that leads to less phosphate loss [2,3]. General symptoms of XLH are scoliosis, lordosis, enlarged wrists, and lateral bowing of the legs [2].

Dental involvement was reported in 23–67% of XLH patients [11]. A severe impact on oral health-related quality of life in XLH has been reported and might be associated with the high number of endodontically affected teeth [12]. In a more recent study, a positive correlation between increasing pulp chamber size and abscess prevalence was shown, and higher serum parathyroid hormone levels and low serum 1,25-dihydroxyvitamin D levels led to more frequent dental abscesses [13].

For a long time, the only treatment option for XLH was multiple daily doses of supplements of inorganic phosphate and vitamin D to avoid decreased serum calcium levels due to phosphate loading and rising parathormone, though this therapy regimen was revealed to have many adverse events [2,14,15,16]. Additionally, there are no changes in dental phenotype and no dental benefits observed as a result of this therapy [14,15,16]. A new option for targeted therapy of XLH that addresses its pathophysiology is Burosumab, a human monoclonal antibody that binds FGF-23 (fibroblast growth factor 23) and inhibits its signaling [17]. So far, the impact of targeted therapy with Burosumab in patients with XLH on the dental manifestation of this disease has not been thoroughly analyzed. Thus, we gathered the existing knowledge on the pathophysiology, dental phenotype and genetic background of XLH and analyzed the different therapeutic options with a focus on targeted therapy and its impact on dental impairment.

## 2. Pathogenesis and Dental Symptoms

The disease results from a disorder of the transepithelial transport of phosphate and hypophosphatemia due to decreased tubular reabsorption of phosphate [8]. Renal phosphate reabsorption through sodium-dependent phosphate transport is impaired in XLH, which results in excessive phosphate excretion. The Hyp-mouse model, in which the 3′-terminus of the PHEX gene is deleted, which leads to insufficient sodium-dependent phosphate co-transport similar to that in XLH in patients, helped with studying whether this defect is caused by an intrinsic renal defect or by hormonal abnormality [9,18]. The currently established observation is that there is a humoral defect in Hyp-mice. Studies suggest that not only renal phosphate transport but also osteoblast mineralization reflects a hormonal imbalance [18].

Onishi et al. (2005) suggest that hyperexpression of osteocalcin, a bone and dentin matrix protein, delays dentin mineralization and can cause hypomineralization of dentin in Hyp-mice [19]. Other studies on Hyp-mice imply that the dentin defects may be related to an intrinsic defect in dentin formation and not directly to XLH [20].

The oral manifestations of XLH can range from minimal to severe [8]. Seow et al. [8] proposed three grades:

- Grade I: minimal or no dental manifestations.
- Grade II: moderate pulp enlargement with a few abscessed teeth.
- Grade III: large pulp chambers and multiple dental abscesses [8].

Delay of tooth eruption, enlarged pulp chambers, and pulpal exposure are typical dental symptoms of this disease [1]. XLH patients usually present with abscesses, fractured teeth, and fistulae without trauma or evident caries [1,2]. This is due to clefts in the dentin up to the dentinoenamel junction covered by a thin layer of enamel and can easily lead to bacterial entry when enamel attrition or minimal chipping occurs [21]. XLH patients can be predisposed to enamel abnormalities [22]. Gingival and mucosal swellings and periapical abscesses are common in both the deciduous and permanent teeth [21].

A higher prevalence of endodontic complications such as apical periodontitis requiring root canal treatment or spontaneous endodontic abscesses has been recorded [23]. Large pulp chambers and extended pulp horns are characteristic even in older patients [23]. Another issue is periodontal bone loss, which is detected in around 60% of patients and requires intensive dental care [24]. Further investigation is needed to determine whether an early onset of periodontal bone loss is a contributing factor.

PHEX (phosphate-regulating endopeptidase homolog, X-linked) deficiency in Hyp-mice has been shown to lead to an altered periodontal phenotype with hypoplasia of the cementum, which could indicate a higher susceptibility to periodontal disease in adult XLH patients [25]. Defects in the cementum, periodontal ligament, or alveolar bone can lead to loss of dental attachment, and a role of FGF-23 in the development of the dentoalveolar complex has been identified [26]. The correlation between XLH and periodontal diseases has not been well defined and needs further investigation [27].

Schwartz et al. (1988) described a higher prevalence of Class III malocclusions in patients with XLH than in healthy relatives, which is probably due to the increased anteroposterior diameter of the skull [28].

The severity of the dental phenotype and the first appearance of oral symptoms have been shown to correlate: the younger the patient with dental abscesses, the more severe the dental manifestation [29].

## 3. Diagnosis

Early diagnosis and treatment can help in maximizing growth and minimizing dental abnormalities [7]. Radiological, biochemical, and clinical aspects should be considered for diagnosis; additionally, in most patients, the PHEX gene mutation can be detected [30].

However, some genetic defects such as large deletions or mosaicism are difficult to identify [30].

Normal serum calcium levels, low serum phosphate levels, and elevated serum alkaline phosphatase activity are suggestive of XLH [9]. Particularly in adults, serum alkaline phosphatase (ALP) activity is not a sensitive parameter with which to monitor the response to therapy [9]. FGF-23 levels can be elevated or inappropriately normal—influenced by phosphate and vitamin D intake—and are most informative in untreated patients [30].

### 3.1. Imaging

Radiographically large pulp chambers, thin dentin and wide root canals are characteristic [3,21]. An in vitro study examining teeth from XLH patients using micro-CT revealed different dentin mineralization patterns compared to teeth from healthy patients [31]. Lower mineral density and porosity in dentin and enamel hypoplasia with thin enamel have been described several times [31].

### 3.2. Histology

Typically, the thin dentin consists of large calcospherites and globules of abnormally calcified dentin that are surrounded by wide irregular zones of interglobular dentin [3]. In some cases, enamel hypoplasia can be found, though an effect on dentin is more common [2,32]. It is characterized by wide zones of irregular dentin, extensive interglobular unmineralized dentin, clefts, and tubular defects that extend to the enamel and allow bacterial invasion into the pulp [2,33]. Secondary and reparative dentin formation might be reduced in XLH, and low serum phosphate levels can impede remineralization [2,21].

Seow et al. divide the histological appearance of dentin into three grades:

- Grade I: <50% of the total dentin thickness is involved, and minimal interglobular spaces are present.
- Grade II: >50% but does not involve entire dentin thickness and moderate interglobular spaces are present.
- Grade III: the entire dentin thickness is involved, and large interglobular spaces are present [8].

The dentin mineralization pattern is disturbed around odontoblasts, and regions of accumulating osteopontin that inhibits mineralization have been identified [25]. Reduced thickness of the cementum with altered periodontal mechanical properties has been detected and could contribute to periodontal defects [27].

## 4. PHEX Gene Mutation and Dental Phenotype

Hypophosphatemia is caused by mutations at the locus HPDR2 on the X chromosome [34]. Not only renal phosphate transport but also the mineralization of bone and teeth is affected [34]. A large number of mutations that inactivate PHEX can cause XLH, and around 300 different mutations in the PHEX gene are recorded [10,30].

The gene responsible for XLH is located on Xp22.1 and is called phosphate-regulating neutral endopeptidase on X-chromosome (PHEX); this gene is expressed in osteoblasts and odontoblasts but not in the kidney [29,35,36,37]. The loss-of-function mutation of PHEX leads to the stimulation of unnecessary proteins such as FGF-23 [10]. An elevated FGF-23 serum concentration in XLH could be a cause of renal and hard-tissue phenotypes in XLH, and a role as an inhibitory factor of mineralization has been described [37]. FGF-23, which is a regulator of phosphate homeostasis, suppresses the expression of type 2a and 2c sodium–phosphate cotransporters in the renal proximal tubules, leading to the inhibition of phosphate resorption and reduced serum phosphate [4,10,38]. Inhibition of 1α-hydroxylase and therefore a reduced 1,25-(OH)_2_ vitamin D concentration are also caused by FGF-23 [38]. This leads to reduced gastrointestinal absorption of phosphate and decreased serum phosphate concentration [39].

FGF-23 is expressed by osteocytes and osteoblasts and is predominantly regulated by serum phosphate and calcitriol [26]. It binds to the FGF receptor–Klotho complex, which is expressed in the kidney and parathyroid glands [4]. Moreover, decreased 1,25-dihydroxyvitamin D levels result in reduced intestinal phosphate resorption, indicating that FGF-23 physiologically regulates phosphate and vitamin D metabolism [4]. FGF-23 seems to play an important role in XLH and its manifestations, but the cause of this is not yet clear [26].

PHEX mutations can inhibit bone mineralization and therefore lead to mineralization defects in bone and dentin via the accumulation of SIBLING (small integrin binding ligand n-linked glycoproteins) protein fragments called acidic serine aspartate-rich MEPE (matrix extracellular phosphoglycoprotein) associated motifs (ASARMs), such as osteopontin and matrix extracellular phosphoglycoprotein [5,40]. Osteopontin, which binds to hydroxyapatite in bone and teeth and inhibits crystal growth, is physiologically degraded by PHEX, but its abundance in dentin correlates with defective mineralization [41].

Holm et al. showed that the mutations are found without specific “hot spots”, which complicates traditional genetic testing methods [36]. A genotype–phenotype correlation could not be drawn, implying that the influence of environmental factors and other genes may also affect the severity of the phenotype [36].

Atypical regulation of 1α-hydroxylase results in inappropriately normal calcitriol levels [42]. Studies show that removal of kidneys did not improve hypophosphatemia, and when a normal kidney was transplanted into a XLH patient, hypophosphatemia reoccurred, which implies that there is no intrinsic defect of inorganic phosphate resorption in the kidneys of XLH patients [42].

Baroncelli et al. (2017) showed that PHEX gene mutation does not correlate with dental phenotype or the severity of the disease [13]. Chaussain-Miller et al. (2007) suggested that the dentin abnormalities might not result from the mutation of PHEX but are probably a consequence of hypophosphatemia and vitamin D deficiency [43]. Dental manifestations of XLH are frequently described, even under systemic treatment [2].

## 5. Dental Management of XLH

Oral hygiene instructions and the regular use of fluorides are the most important preventive strategies [2]. Dental management consists of restorative and vital pulp therapy to prevent pulp exposure [2]. Composite resins can be used; for long-lasting prevention, crowns requiring minimal preparation, such as stainless steel crowns, are recommended [44]. Seow et al. suggest replacing steel crowns with gold or porcelain crowns in adulthood [44]. Advances in adhesive dentistry open up new modern and minimally invasive therapy options, for example, ceramic onlay veneers that require minimal substance reduction and adhesive bonding. Therefore, the use of steel crowns on permanent teeth should be restricted.

However, the use of acid materials such as sealants is controversial because these materials may leak through the enamel to the pulp chamber; especially, prolonged etching is related to an increased risk of pulp irritation [9,29]. Other studies recommend fissure sealants as soon as the teeth erupt because these sealants can prevent bacterial invasion of enamel microfractures [38]. Splints can be another helpful tool to reduce attrition and prevent the teeth from being damaged [21]. Andersen et al. (2011) point out that extensive dental care led to the maintenance of a higher number of teeth [45].

The apical periodontitis caused by enamel and dentin alterations requires endodontic treatment; therefore, the prevalence of endodontically treated teeth is higher in patients with XLH and increases significantly with age [12,45,46]. Long-term studies are needed to assess the prognosis of endodontically treated teeth in XLH.

Hanisch et al. (2019) show that patients with oral involvement had worse oral-health-related quality of life values than those with no oral symptoms, which makes regular dental evaluations even more important [46]. Treatment of XLH soon after birth can potentially improve the development of permanent teeth, which form after birth [24,38].

The Asia-Pacific Consensus Recommendations for multidisciplinary referral of patients with XLH include regular screenings at the dentist or endodontist for prevention and timely treatment of dental conditions such as periodontitis or dental abscess [47]. The recommended dental follow-up monitoring for children, adolescents and adults with XLH is twice yearly [47].

## 6. The Impact of Therapy on Dental Impairment

For years, daily doses of supplements of inorganic phosphate and vitamin D have been the gold standard in the therapy of XLH [2]. Renal failure, hypercalcemia, and nephrocalcinosis are some of the side effects of this therapy regimen [14,15,16]. These complications could cause chronic kidney disease; therefore, monitoring of serum and uric parameters is necessary to avoid these side effects [48]. Conventional therapy has additional complications such as gastrointestinal side effects and ectopic calcification [48]. Another complication of therapy with high-dose phosphate is hyperparathyroidism, resulting in hypercalcemia and renal phosphate loss [16]. It is described that the parathyroid tends to become hyperplastic in patients with XLH and should therefore be monitored carefully during therapy [9].

Up to now, there are no changes in dental phenotype and no dental benefits observed with this therapy, which makes dental prophylactic procedures essential [14,15,16]. Furthermore, this indicates that the dentin deformities are not solely caused by phosphate deficiency during odontogenesis [15]. Some studies have seen beneficial effects of phosphate substitution and vitamin D supplements on oral health, especially when medical interventions started in early childhood [11,24,29,49].

Coyac et al. showed abnormal mineralization in XLH cell cultures independent of hypophosphatemia but possibly occurring through the accumulation of osteopontin and changes in DMP1 and MEPE cleavage [50]. In particular, elevated FGF-23 levels, which cause renal phosphate loss, lead to defects in mineralized tissue and should be addressed with therapy [50]. Mineralization defects in the teeth of Hyp-mice seemed to be persistent even when hypophosphatemia was corrected and FGF23 was ablated [27]. The exact pathological mechanism of the dental defects in XLH has not been clarified yet [27].

The symptomatic therapy of XLH with supplements of phosphate and vitamin D is not favorable due to adherence issues and adverse side effects such as nephrocalcinosis, hyperparathyroidism, and chronic kidney disease [17]. Additionally, conventional therapy with supplements of phosphate and vitamin D might have limited effects because of further stimulation of FGF-23 levels and renal phosphate wasting, resulting in a vicious circle [30].

The monoclonal antibody Burosumab, which binds FGF-23 and limits its signaling, offers a new treatment for patients with XLH [17]. Treatment with Burosumab increases serum phosphate and 1,25(OH)_2_D levels. Double-blind, placebo-controlled studies showed that the drug exerted promising effects on bone healing by normalizing phosphate homeostasis and had an acceptable safety profile [17]. Especially for young children suffering from XLH, Burosumab showed promising effects and significantly improved the symptoms [51]. Additionally, treatment with Burosumab produced greater improvements in phosphate metabolism and osteomalacia healing than conventional therapy [52]. Burosumab is approved in the United States and in Europe for the treatment of XLH in adults and children aged 6 months and older [5,7]. Burosumab is injected under the skin of the upper arm, abdomen, buttocks or thigh. The dosage is based on body weight and is slowly increased to a maximum of 90 mg.

In children and adolescents aged 1 to 17 years, the therapy is administered every 2 weeks; in adults, it is administered every 4 weeks. During therapy with Burosumab, the serum concentration of phosphate reflects the efficiency of this treatment and should be monitored [30].

Improvements in pain, stiffness, fracture healing, and physical functions during treatment with Burosumab have been observed [39]. The mild to moderately severe adverse events such as headache, back pain, and injection-related reactions imply well-tolerated therapy with Burosumab [39]. The most common side effects were diarrhea, arthralgia, and reactions at the injection site [53]. Mean serum calcium concentration remained normal, and no evidence of worsening nephrocalcinosis (as observed in conventional therapy) could be found [53].

A change in the radiographic severity of skeletal impairment by XLH was detected 40 and 64 weeks after starting treatment with Burosumab [5]. Boukpessi et al. reported that current therapies for XLH addressing serum phosphate levels and 1,25-dihydroxyvitamin D levels might not influence the osteopontin-induced hypomineralization defect locally in the extracellular matrix of bone and teeth [41]. Burosumab addresses the systemic hypophosphatemia mediated by FGF-23, but the osteopontin-mediated local hypomineralization defect has not yet been targeted [41].

Patients who received Burosumab had a prevalence of 31 to 54% of dental complications such as caries and submucous abscesses [54]. In a phase 3 trial of a study comparing the treatment of XLH with Burosumab to oral phosphate and calcitriol, dental complications were detected more often in patients receiving Burosumab than in those who received oral phosphate and calcitriol [54]. It has been suggested that other biologically active peptides such as osteopontin and MEPE, which are elevated in XLH and are not targeted by Burosumab, might be causative of these dental complications [54,55].

Gadion et al. recently investigated the impact of Burosumab on the mineralization of dental tissues and showed a reduced number of dental abscesses in XLH children treated with Burosumab [49] (Table 1). The authors hypothesized that the immune response may be improved by decreasing FGF-23 serum levels with Burosumab treatment, which may reduce the occurrence of dental abscesses, since high FGF-23 serum levels have been shown to correlate with an increased risk of infection [49].

Early treatment with Burosumab in Hyp-mice showed increases in predentin formation, dentin mineralization, and dentin crown volume [49]. Whether increased FGF-23 secretion directly contributes to mineralization defects in dentin remains unknown [49].

Additionally, the age at which treatment starts is an important predictive factor and has not yet been evaluated for dental involvement, but it is expected that later treatment has less effect on reducing dental abscesses [49]. However, Gadion et al. showed that treatment with Burosumab at a later age decreased the incidence of dental abscesses compared to conventional therapy with phosphate supplements and calcitriol [49].

Ward et al. reported that Burosumab is more preventive than conventional therapy against the development of dental abscesses in patients under the age of 5 years, though in the age group of 5 to 12 years, dental abscesses occurred in 53% of patients [52,56]. Further investigation is needed to explore whether there is a window of opportunity in children to promote dentin mineralization [52].

A study by Kato et al. supports the hypothesis that early treatment before the age of 5 years with phosphate supplements and vitamin D may have a preventive effect on dental complications [56].

Brener et al. (2022) explored the effect of Burosumab on the dental health, dentition, and tooth morphology of ten children with XLH in a prospective case–control study [57]. They included children 8.8 ± 3.8 years old that who were diagnosed at a mean age of 1.9 ± 1.3 years and had been treated with conventional therapy for 7.0 ± 4.2 years [57]. Clinical, radiographic, and laboratory evaluations of dental status were performed during the initiation of Burosumab therapy and after one and three years. Enlarged pulp chambers persisted after treatment with Burosumab, as demonstrated by larger pulp-to-coronal height and width ratios in XLH patients than in healthy controls [57]. Dental abscesses were significantly decreased during treatment with Burosumab. However, the authors indicate that this might be because of increased awareness of dental hygiene [57]. Brener et al. (2022) concluded that osteopontin accumulating in the dentin of XLH patients and not being targeted by Burosumab may contribute to the persistence of pathologic dental morphology [57]. The mean age of the patients in this study treated with Burosumab is higher than in the study of Ward et al. [52]; therefore, the treatment might not have had a significant influence on dental mineralization and morphology.

Linglart et al. (2022) reported the results after three years of Burosumab therapy and confirmed that this treatment modality was well tolerated and improved the symptoms of XLH significantly [58]. Whether mineralization of dentin improves with the normalization of phosphate levels remains unclear and needs further investigation [52]. Additionally, Burosumab’s long-term effects on dental anomalies have not yet been examined [7].

## 7. Conclusions

In conclusion, due to the severe dental complications caused by XLH, consistent diagnosis and early treatment are crucial. Continuous conservative treatment with phosphate and calcitriol supplements to stabilize serum phosphate levels did not yield significantly reduced dental comorbidities in XLH. In contrast, targeted therapy with Burosumab represents a novel therapeutic alternative and produces considerable improvement in the management of pain, fracture incidence, and physical functioning. Decreased incidence of dental abscesses and increased dentin crown volume during treatment with Burosumab have been described as improvements. Other studies have observed a higher rate of dental complications with Burosumab than with conservative treatment. It remains unclear whether normalizing phosphate levels improves dental impairment and serum phosphate levels directly influence dental development or whether other unknown mechanisms are responsible for the dental phenotype. Attention should also be drawn to the impact of biologically active peptides that are not targeted by Burosumab, such as osteopontin and MEPE, but might contribute to the dental manifestation of this disease and could be possible targets for future progression in dental therapy. Further long-term investigation of the impact of Burosumab on dental complications in XLH is required.

## Figures and Tables

**Table 1:** A list of clinical trials comparing conventional therapy with oral phosphate and calcitriol substitution to Burosumab treatment and its influence on dental impairment.

Authors (Year)	# of XLH Patients	Control Group	Duration	Dental Outcome
Imel EA et al. (2019) [55]	61	Conventional therapy	64 weeks	Higher prevalence of dental abscesses in the Burosumab group
Gadion M et al. (2022) [49]	71	Conventional therapy	≥1 year	Decreased mean number of dental abscesses in the Burosumab group, caries’ prevalence same in both groups
Ward LM et al. (2022) [52]	61	Conventional therapy	64 weeks	No dental abscesses in patients under the age of five in the Burosumab group, dental abscesses in 53% of older children in the Burosumab group

## Data Availability

The data used to support the content of this study are included within the article.
